# Impact of Body Weight on Sedation-Related Outcomes in Complex Electrophysiological Interventions

**DOI:** 10.3390/healthcare14040517

**Published:** 2026-02-18

**Authors:** Lyuboslav Katov, Celine Dupree, Yannick Teumer, Alexandra Buss, Federica Diofano, Carlo Bothner, Wolfgang Öchsner, Wolfgang Rottbauer, Karolina Weinmann-Emhardt

**Affiliations:** 1 Department of Cardiology, Ulm University Heart Center, Albert-Einstein-Allee 23, 89081 Ulm, Germany; lyuboslav.katov@uniklinik-ulm.de (L.K.); celine.dupree@uni-ulm.de (C.D.); yannick.teumer@uniklinik-ulm.de (Y.T.); alexandra.buss@uniklinik-ulm.de (A.B.); federica.diofano@uniklinik-ulm.de (F.D.); carlo.bothner@uniklinik-ulm.de (C.B.); wolfgang.rottbauer@uniklinik-ulm.de (W.R.); 2 Department of Anesthesiology and Intensive Care Medicine, Ulm University Medical Center, Albert-Einstein-Allee 23, 89081 Ulm, Germany; wolfgang.oechsner@uniklinik-ulm.de

**Keywords:** deep sedation, electrophysiological procedures, body weight, BMI, blood gas analysis, hemodynamic outcomes, respiratory complications

## Abstract

Background: Higher body mass index (BMI) is associated with a greater burden of cardiometabolic comorbidities that may potentially increase the risk of sedation-related complications. However, the impact of BMI on sedation safety during complex electrophysiological procedures (CEPs) remains uncertain. Methods: In this study conducted at Ulm University Heart Center, patients undergoing CEPs were stratified into three BMI groups: normal weight, overweight, and obesity. Primary and secondary endpoints were analyzed using univariable and multivariable logistic regression analyses. The primary composite endpoint (PCE) was the occurrence of sedation-related complications, defined as oxygen saturation below 90% combined with abnormal peripheral venous blood gas results—specifically, a venous carbon dioxide level exceeding 70 mmHg, an increase of more than 30% from baseline, or a pH drop below 7.25. Secondary endpoints included sedative and opioid requirements as well as occurrence of individual components of the PCE. Results: A total of 726 patients were included, with 299 (42.3%) being female. The study population comprised 236 patients (32.5%) of normal weight, 265 (36.5%) overweight, and 225 (31.0%) obese. Patients with higher BMI presented with a greater burden of comorbidities and lower baseline oxygen saturation at the start of the procedure. While absolute sedative and opioid doses remained stable or even increased with BMI, relative doses (mg/kg) were higher in normal-weight patients. No significant differences were observed between BMI groups for either the primary or secondary endpoints. Female sex emerged as an independent predictor of adverse sedation events, with a higher incidence of reaching the PCE (*p* = 0.046, OR 1.411). Conclusions: BMI alone was not associated with an increased risk of sedation-related complications during CEPs. Despite higher absolute drug requirements and a greater comorbidity burden in overweight and obese patients, procedural safety was comparable across all BMI categories. These findings emphasize that individualized sedation protocols, embedded within standardized monitoring frameworks, are essential to ensure safe and effective sedation in diverse patient populations.

## 1. Introduction

Catheter ablation is a frequently utilized, minimally invasive procedure aimed at treating cardiac arrhythmias [1,2]. Sedation is a key component of electrophysiological interventions, as it facilitates patient comfort by ensuring stillness, adequate pain control, and unconsciousness throughout the procedure [3,4]. Despite its respiratory depressant properties, sedation must be carefully balanced to maintain stable cardiovascular function and appropriate gas exchange. Meticulous sedation strategies are crucial to preventing adverse effects such as oxygen desaturation, elevated carbon dioxide levels, and hemodynamic disturbances, thereby promoting procedural success and patient safety. There are various sedation strategies employed during electrophysiological procedures, ranging from deep sedation with preserved spontaneous respiration to general anesthesia with complete loss of consciousness and airway management through endotracheal intubation. The choice of sedation technique often varies depending on the country, institutional standards, available resources, and the expertise of the medical team.

Recent research has highlighted that patient-specific factors—particularly body weight—can significantly influence sedation-associated outcomes during electrophysiological procedures. It is well known that obesity is a growing global epidemic affecting anesthesiologists, surgeons, and intensive care specialists [5]. Since 1975, the number of obese people has almost tripled [6]. In Europe and North America, more than 60% of adults are overweight and nearly one third of them are obese [7]. Obese individuals often exhibit altered pharmacokinetics and pharmacodynamics due to increased adipose tissue, changes in cardiac output, and reduced pulmonary compliance, all of which may impact the metabolism and distribution of sedative agents [8,9,10]. As a result, patients with higher body mass indices (BMI) are at greater risk for sedation-related respiratory complications, including hypoventilation, airway obstruction, and prolonged recovery times [11]. Conversely, underweight patients may also present unique challenges, including altered sensitivity to sedative agents and reduced physiological reserves, which may predispose them to hypotension or rapid desaturation [12,13].

Furthermore, studies have demonstrated that blood gas abnormalities, including hypercapnia and hypoxemia, occur more frequently in overweight and obese patients undergoing deep sedation, which may compromise hemodynamic stability and procedural safety if not promptly addressed [14,15]. These weight-related variations necessitate individualized sedation protocols that account for both the pharmacological and physiological nuances of different body types. Therefore, stratifying sedation management by patient weight may enhance the safety and effectiveness of electrophysiological procedures, while minimizing the risk of adverse events. This study aims to evaluate the impact of body weight differences on electrophysiological procedures performed under deep sedation.

## 2. Materials and Methods

### 2.1. Study Design and Population

This prospective clinical trial enrolled consecutive adult patients aged 18 years and older who were scheduled to undergo electrophysiological procedures under deep sedation at the University Heart Center Ulm between August 2019 and October 2023. Both male and female patients were eligible. The study population included individuals requiring electrophysiological interventions such as cryoballoon pulmonary vein isolation and combined atrial and ventricular 3D electroanatomical mapping. Patients were allocated into three groups according to body weight status, based on BMI: normal weight (BMI < 25), overweight (BMI 25–30), and obese (BMI > 30). Written informed consent was obtained from all participants prior to the intervention. Patients were excluded if they declined to provide consent or if their procedures were performed under mild instead of deep sedation. The trial protocol was approved by the Ethics Committee of the University of Ulm and was conducted in accordance with the ethical principles of the Declaration of Helsinki (protocol code 324/16, 12 October 2016). This study was registered in the German Clinical Trials Register (register id: DRKS00013013, register date: 12 May 2017).

### 2.2. Monitoring Setup and Sedation

The standard monitoring described in this study is consistent with previously published recommendations for procedural sedation performed by non-anesthesiologists [3,16]. During the procedure, patients underwent continuous monitoring, including electrocardiography, heart rate assessment, and pulse oximetry to evaluate oxygen saturation (SpO_2_). Non-invasive blood pressure was measured and documented at three-minute intervals, while peripheral venous blood gas samples were obtained every 30 min. In a subset of patients, transcutaneous carbon dioxide levels were monitored using a forehead-mounted electrochemical sensor (TCM400, Radiometer^®^, CPH, Denmark), in accordance with a previously established block randomization protocol [17].

A 5 mg bolus of midazolam was administered at the beginning of the procedure to provide anxiolysis. This was followed by a continuous propofol infusion to maintain a level of deep sedation. Prior to the start of cryoballoon pulmonary vein isolation, fentanyl was given, while patients undergoing interventions involving 3D electroanatomical mapping received a continuous infusion of remifentanil instead. Airway patency was supported using Wendel and/or Guedel airway devices, and additional oxygen was supplied via a face mask with reservoir bag. A sedation assistant was responsible for documenting the entire sedation process, including airway management steps, peripheral venous blood gas measurements, and the exact timing and dosages of all administered medications. Transesophageal echocardiography was not routinely utilized during the procedures. Its use was generally limited to the pre-procedural setting, primarily to rule out intracardiac thrombi in patients with inadequate oral anticoagulation.

### 2.3. Primary and Secondary Endpoints

SpO_2_ values falling below 90%, combined with abnormal peripheral venous blood gas results—defined as a partial pressure of venous carbon dioxide (PvCO_2_) level exceeding 70 mmHg, a PvCO_2_ increase of more than 30% from baseline, or a pH drop below 7.25—were designated as the primary composite endpoint. The secondary endpoints included each individual parameter of the primary outcome, as well as hypotensive events, defined by a systolic blood pressure under 80 mmHg or a mean arterial pressure below 65 mmHg. In addition, variations in the administration of sedatives and opioids were evaluated, with opioid doses standardized to morphine equivalents.

The primary and secondary endpoints were continuously monitored during the electrophysiological procedure under deep sedation and were intended to reflect clinically relevant physiological disturbances necessitating immediate clinical intervention. These endpoints did not encompass downstream outcomes such as procedure interruption, anesthesiology consultation, or intensive care admission.

### 2.4. Statistical Analysis

Statistical analyses were performed using SPSS Statistics software (version 29, IBM, Armonk, NY, USA). Categorical variables were analyzed using the chi-square test, while continuous variables were assessed using the Mann–Whitney U test or the Kruskal–Wallis test, as appropriate. Continuous variables are presented as median and interquartile range (IQR), with mean ± standard deviation (SD) additionally reported in brackets. To explore predictors of improved clinical outcomes, logistic regression analyses were conducted for both primary and secondary endpoints. Differences in anesthetic dosing were evaluated using linear regression. To evaluate potential effect modification, dummy-coded BMI categories and their interaction terms with sex were included in an additional multivariable logistic regression model. Detailed information on variable coding in the multivariable analyses is provided in Supplementary Table S1. Model calibration was assessed using the Hosmer–Lemeshow goodness-of-fit test, discrimination using the area under the Receiver operating characteristic (ROC) curve, and multicollinearity using variance inflation factors (VIF). A *p*-value of less than 0.05 was interpreted as statistically significant.

The present study represents a secondary analysis of a previously conducted prospective clinical trial. The original sample size calculation was performed for the primary study endpoint and has been reported previously [17]. No separate sample size calculation was performed for the BMI-based analyses within the overall prospectively enrolled cohort; therefore, all analyses should be considered exploratory in nature.

## 3. Results

### 3.1. Patients’ Characteristics

**Figure 1:**
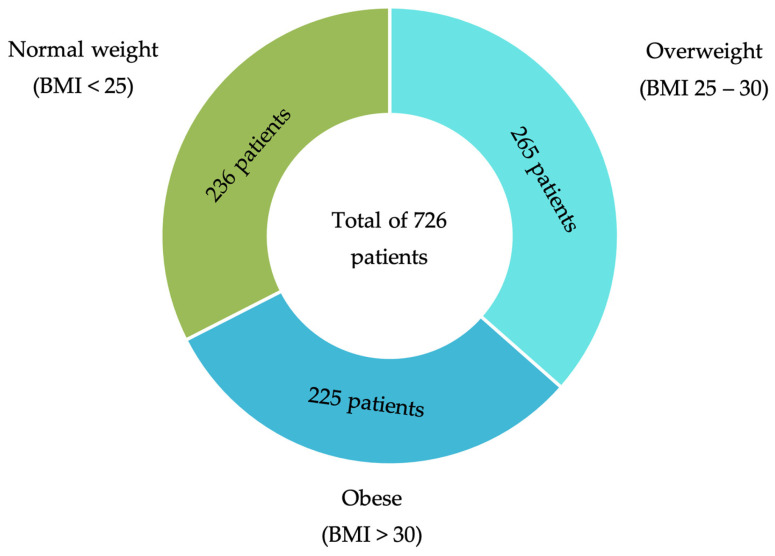
Depiction of the patient’s distribution across the three BMI groups. BMI, body mass index.

A total of 726 patients were enrolled in the trial, comprising 427 men (58.8%) and 299 women (41.2%). Based on BMI, 236 patients (32.5%) were classified as having normal weight, 265 (36.5%) as overweight, and 225 (31.0%) as obese (Figure 1).

The overall mean age was 67.0 ± 11.5 years. Patients with normal weight were significantly older than those in the overweight and obese groups (*p* < 0.001). Compared to normal-weight individuals, overweight and obese patients showed a significantly higher prevalence of arterial hypertension (*p* < 0.001), diabetes mellitus (*p* < 0.001), and obstructive sleep apnea syndrome (OSAS) (*p* < 0.001). The distribution of procedure types, including cryoballoon ablation and 3D electroanatomical mapping, did not differ significantly across BMI categories (*p* = 0.743). No significant differences were observed between the groups with respect to the remaining clinical variables (Table 1).

### 3.2. Treatment Characteristics

The mean procedure duration was 165.6 ± 76.9 min, with no significant differences between the groups (*p* = 0.295). Cardioversion was performed in nearly half of the procedures. The mean initial O_2_ starting dose was 6.3 ± 2.5 L/min. Compared to normal-weight patients, obese patients required a significantly higher O_2_ starting dose (*p* = 0.007). The mean baseline oxygen saturation was 97.6 ± 2.8%, and was lower in obese patients compared to normal-weight patients (*p* = 0.050). Comparison of heart rate, systolic and mean blood pressures revealed no significant differences between the three subgroups. An overview of all treatment characteristics is presented in Table 2.

### 3.3. Primary Composite Endpoint

**Figure 2:**
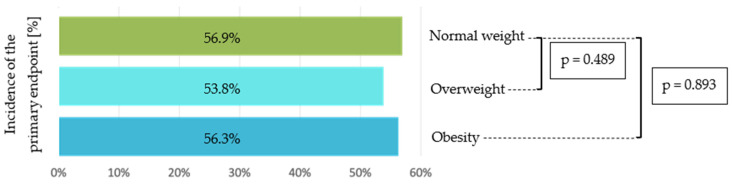
Comparison of primary composite endpoint outcomes between normal weight, overweight and obese patients.

The primary composite endpoint, defined as the combination of SpO_2_ falling below 90% and the occurrence of pathological changes in venous blood gas analysis, was observed in 390 patients (55.6%) across all treated patients. In the normal-weight group 123 patients (56.9%) reached the primary endpoint, while in the overweight group 142 patients (53.8%) and in the obese group 125 patients (56.3%) reached the primary composite endpoint. Statistical analysis revealed no significant differences between the three groups (normal vs. overweight *p* = 0.489 and normal vs. obese *p* = 0.893) (Figure 2).

Multivariable logistic regression identified female sex as an independent predictor of the primary endpoint (OR 1.41, 95% CI 1.01–1.98, *p* = 0.046). Additional multivariable analysis including interaction terms between BMI category and sex did not demonstrate a significant interaction effect (*p* = 0.734 for overweight × sex and *p* = 0.383 for obesity × sex). Diabetes mellitus showed a trend towards increased risk without reaching statistical significance (OR 1.50, 95% CI 0.97–2.33, *p* = 0.070). Higher BMI and the other comorbidities included in the multivariable model showed no significant association with the primary composite endpoint (Table 3).

**Figure 3:**
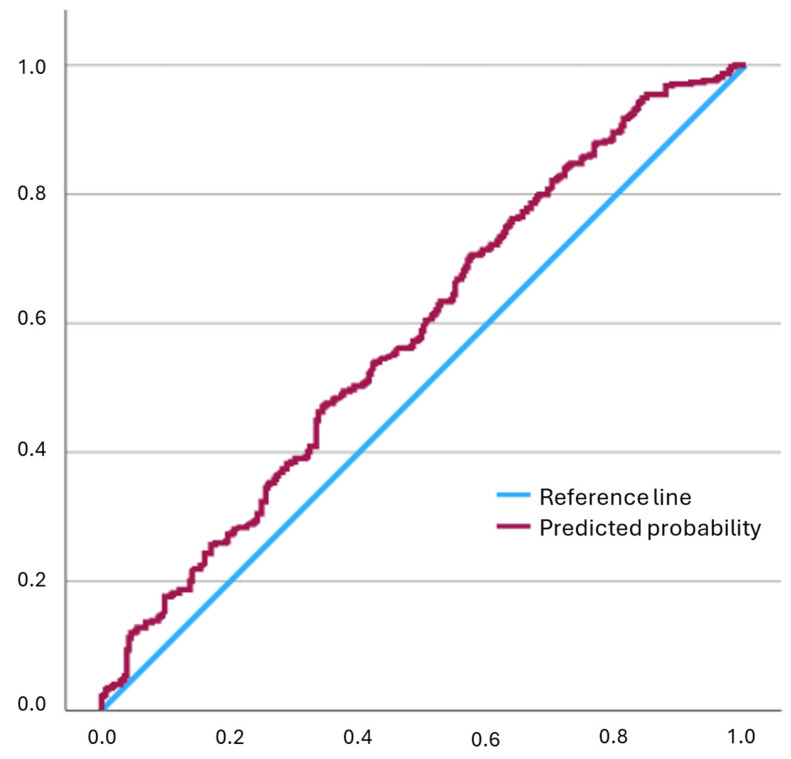
Receiver operating characteristic (ROC) curve of the logistic regression model for the prediction of the primary endpoint based on predicted probabilities. The blue diagonal line represents the reference line, and the red line represents the ROC curve of the model.

The logistic regression model demonstrated adequate calibration (Hosmer–Lemeshow goodness-of-fit test, *p* = 0.221) and limited discrimination (AUC = 0.6) (Figure 3). No relevant multicollinearity was observed, as all VIF were below the accepted threshold of 2.

### 3.4. Secondary Endpoints

Analysis of respiratory and hemodynamic endpoints revealed no significant differences among the BMI groups. The incidence of oxygen desaturation below 90% was comparable across groups (*p* = 0.575). Severe hypercapnia (PvCO_2_ >70 mmHg) was infrequent (4% of the overall cohort) and evenly distributed (*p* = 0.878). Similarly, venous pH < 7.25 showed no group-related variation (*p* = 0.774). A systolic blood pressure < 80 mmHg occurred in 278 patients (38.3%), and a mean arterial pressure < 65 mmHg in 372 patients (52.1%), without intergroup differences. Detailed overview of the endpoints between the groups is presented in Table 4.

**Figure 4:**
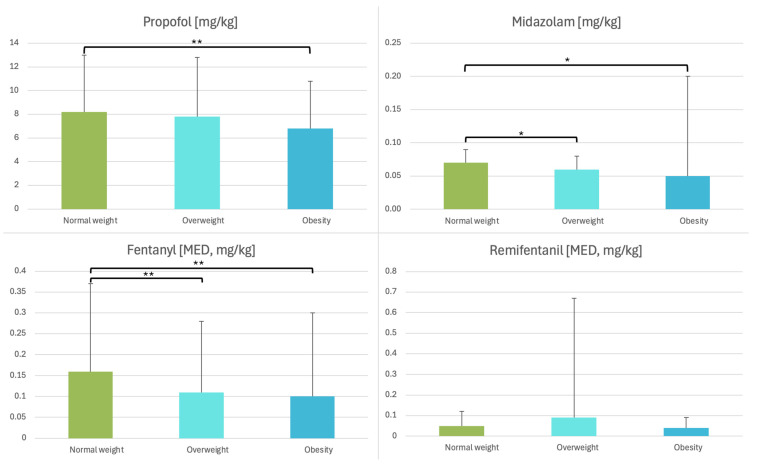
Comparison of drug dosages in mg/kg between the groups. Error bars represent standard deviation. MED, morphine equivalent dose. * *p* < 0.001, ** *p* ≤ 0.005.

Significant differences were observed in the drug administration. Patients with elevated BMI received lower weight-adjusted doses of midazolam (*p* < 0.001), propofol (*p* = 0.008), and fentanyl (*p* = 0.004) (Figure 4).

## 4. Discussion

To the best of our knowledge, evidence regarding procedural characteristics and safety in overweight and obese patients undergoing electrophysiological procedures under moderate to deep sedation remains scarce. Most of the available data addressing sedation in obese populations originate from studies in endoscopy and bronchoscopy, with only limited applicability to electrophysiology [11,18,19]. Given the increasing prevalence of obesity and its well-documented impact on airway management, ventilation, and hemodynamic stability, further research is warranted [20]. The present study aims to evaluate the influence of different weight categories on sedation-related outcomes in electrophysiological procedures performed under sedation. Particular attention is directed toward respiratory and hemodynamic parameters, which are known to be adversely affected by obesity and may directly impact procedural safety [21].

Approximately two-thirds of the patients in this study had an elevated BMI, consistent with prior reports [22]. Increased BMI is frequently associated with comorbidities such as arterial hypertension and diabetes mellitus, even at a younger age, as demonstrated in our cohort. Both conditions predispose to hemodynamic instability and may complicate sedation management [23]. Moreover, obesity is linked to characteristic physiological and anatomical alterations, including a reduction in functional residual capacity, a higher risk of upper airway obstruction, and an increased prevalence of OSAS [7,10,24]. These factors likely contributed to the lower baseline oxygen saturation and the higher oxygen flow requirements observed at the beginning of the procedure in our study. Despite this higher baseline risk profile, no increase in sedation-related complications was observed across BMI categories. This apparent discrepancy is likely explained by standardized sedation protocols, early escalation of oxygen therapy, and close cardiorespiratory monitoring, which may have mitigated obesity-related physiological risks before clinically relevant adverse events occurred [3,16].

The primary composite endpoint, defined as oxygen desaturation below 90% combined with pathological changes in venous blood gas analysis, was achieved in more than half of all patients, regardless of BMI. This finding is consistent with the results reported by Maslova et al. and Tabaja et al., who likewise observed no increase in periprocedural risk during catheter ablation among patients with elevated BMI [25,26]. Moreover, no significant differences were observed between the three BMI groups regarding the secondary endpoints, such as hypercapnia, respiratory acidosis, or hypotension. One possible explanation is that periprocedural management strategies—including careful patient selection, standardized sedation protocols, and close respiratory and hemodynamic monitoring—may mitigate the physiological risks typically associated with elevated BMI. In addition, advances in ablation technology and perioperative care appear to offset potential challenges such as difficult airway management, altered drug pharmacokinetics, and impaired respiratory mechanics, resulting in comparable short-term safety outcomes across BMI categories. The primary composite endpoint was defined as the occurrence of either oxygen desaturation below 90% or pathological venous blood gas parameters. Fulfillment of any single criterion was sufficient to classify an event as positive. This definition was chosen to ensure comprehensive detection of clinically relevant respiratory compromise during deep sedation, capturing both acute hypoxemia and hypercapnic deterioration. All individual components were additionally analyzed separately and did not demonstrate significant differences across BMI categories.

Sedative dosing demonstrated a balancing effect across BMI categories. Although absolute sedative requirements increased with higher BMI, the relative dose (mg/kg) was higher in normal-weight patients. Dosing was not proportional to total body mass, as this would have increased the risk of overdose in obese individuals, as shown by Ingrande and Lemmens [27]. Both midazolam and propofol are highly lipophilic, resulting in an expanded volume of distribution with increasing fat mass and, consequently, higher absolute doses [28]. However, their pharmacodynamic effects and clearance were primarily related to lean body mass rather than adipose tissue [27,28]. Therefore, the clinically required dose per kilogram did not rise proportionally with BMI. Despite the higher absolute propofol doses in obese patients, normal-weight patients received a higher relative dose, resulting in a comparable overall sedation load across BMI groups [29,30].

Our analysis further highlighted gender-related differences in sedation outcomes. Female sex emerged as an independent predictor of adverse sedation events, consistent with previous reports suggesting that women may exhibit greater sensitivity to sedatives and a narrower therapeutic window [31,32]. Importantly, the additional multivariable analysis including interaction terms between BMI category and sex did not demonstrate a significant interaction effect, indicating that the observed association between female sex and the primary endpoint was independent of BMI category. Recently, gender differences in sedation practice and safety have been described, with women showing increased susceptibility to hypoxemia and hemodynamic instability compared to men [33]. These findings underline the importance of considering sex as a biological variable when tailoring sedative regimens. While diabetes mellitus demonstrated a tendency toward higher risk, this association did not reach statistical significance, and neither BMI nor other comorbidities independently influenced the primary composite endpoint. Overall, these observations suggest that patient sex may represent a more critical determinant of sedation safety than traditional risk factors such as obesity, reinforcing the need for individualized approaches in procedural sedation.

In summary, although obesity is associated with an increased prevalence of cardiometabolic comorbidities and anatomical risk factors, our study demonstrated that these were not linked to a higher incidence of sedation-related complications. During complex electrophysiological procedures, individualized sedation and standardized monitoring protocols are essential to ensure early recognition and management of sedation-related adverse effects, including cardiorespiratory depression associated with higher sedative and opioid doses.

### Limitations

Several limitations of the present study should be acknowledged. Obesity was analyzed as a single category defined by a BMI ≥ 30 kg/m^2^. Although a more granular stratification according to World Health Organization obesity classes (30–34.9, 35–39.9, ≥ 40 kg/m^2^) could provide additional insights, the study was not powered to detect differences across obesity subclasses, and further subdivision would have resulted in small subgroup sizes with limited statistical reliability. Therefore, a pragmatic BMI-based classification was chosen to reflect routine clinical practice.

While OSAS was included as a comorbidity, a more detailed stratification based on OSAS severity or validated screening tools such as STOP-Bang scores was not performed. Such an approach may further refine risk assessment but was beyond the scope of the present study. Future prospective investigations specifically designed to address obesity subclasses and OSAS severity are warranted to better characterize high-risk patient subgroups.

## 5. Conclusions

Our findings indicate that BMI alone is not a decisive factor for sedation safety in electrophysiological procedures. Despite higher absolute drug requirements and a greater burden of comorbidities in overweight and obese patients, complication rates did not differ from those in normal-weight individuals. These results emphasize that, within a standardized framework, individualized monitoring and sedation protocols are essential to ensure procedural safety across all BMI categories.

## Figures and Tables

**Table 1:** Patients’ characteristics.

	Total (*n* = 726)	Normal (*n* = 236)	Overweight (*n* = 265)	Obesity (*n* = 225)	*p*-Value (Between Groups)
Age [years], median (IQR) [mean ± SD]	68.0 (61.0; 76.0) [67.0 ± 11.5]	72.0 (63.0; 78.0) [69.2 ± 12.7]	68.0 (59.0; 75.0) [66.4 ± 11.5]	66.0 (58.2; 73.0) [65.7 ± 9.9]	<0.001
Body Mass Index, median (IQR) [mean ± SD]	27.5 (24.5; 31.1) [28.5 ± 5.6]	23.3 (22.1; 24.2) [23.0 ± 2.3]	27.4 (26.2; 28.4) [27.4 ± 1.3]	33.3 (31.4; 37.1) [35.0 ± 4.9]	<0.001
Female, n (%)	299 (41.2)	103 (43.6)	96 (36.2)	100 (44.4)	0.033
Arterial hypertension, n (%)	523 (72.0)	145 (61.4)	188 (70.9)	190 (84.4)	<0.001
Hyperlipoproteinemia, n (%)	434 (59.8)	123 (52.1)	166 (62.6)	145 (64.4)	0.239
Diabetes mellitus, n (%)	117 (16.1)	21 (8.9)	32 (12.1)	64 (28.4)	<0.001
(Former) Smoker, n (%)	190 (26.2)	51 (21.6)	76 (28.7)	63 (28.0)	0.418
Coronary artery disease, n (%)	257 (35.4)	83 (35.2)	94 (35.5)	80 (35.6)	0.784
Reduced LVEF, n (%)	304 (41.9)	84 (35.6)	120 (45.3)	100 (44.4)	0.265
COPD, n (%)	41 (5.6)	12 (5.1)	14 (5.3)	15 (6.7)	0.791
Asthma, n (%)	22 (3.0)	5 (2.1)	8 (3.0)	9 (4.0)	0.587
OSAS, n (%)	50 (6.9)	6 (2.5)	13 (4.9)	31 (13.8)	<0.001
Pulmonary hypertension, n (%)	21 (2.9)	7 (3.0)	7 (2.6)	7 (3.1)	0.922

COPD, chronic obstructive pulmonary disease; IQR, interquartile range; LVEF, left ventricular ejection fraction; OSAS, obstructive sleep apnea syndrome; SD, standard deviation.

**Table 2:** Treatment characteristics.

	Total (*n =* 726)	Normal (*n* = 236)	Overweight (*n* = 265)	Obesity (*n =* 225)	*p*-Value (Between Groups)
Procedure duration (min), median (IQR) [mean ± SD]	150.0 (110.0; 205.7) [165.6 ± 76.9]	134.0 (107.0; 204.0) [162.0 ± 79.5]	150.5 (111.0; 210.0) [166.3 ± 74.4]	156.0 (118.0; 207.0) [168.3 ± 77.6]	0.295
Intraprocedural cardioversion, n (%)	331.0 (45.6)	100 (42.4)	123.0 (46.4)	108.0 (48.0)	0.987
Initial oxygen flow (L/min), median (IQR) [mean ± SD]	8.0 (4.0; 8.0) [6.3 ± 2.5]	8.0 (3.0; 8.0) [6.1 ± 2.6]	8.0 (4.0; 8.0) [6.2 ± 2.5]	8.0 (6.0; 8.0) [6.8 ± 2.3]	0.007
Baseline oxygen saturation (%),median (IQR) [mean ± SD]	98.0 (97.0; 99.0) [97.6 ± 2.8]	99.0 (97.0; 100.0) [97.8 ± 3.2]	98.0 (97.0; 99.0) [97.7 ± 2.3]	98.0 (97.0; 99.0) [97.4 ± 2.8]	0.050
Baseline heart rate (bpm), median (IQR) [mean ± SD]	77.0 (62.0; 94.0) [81.6 ± 26.4]	77.0 (63.5; 94.0) [82.1 ± 25.7]	77.0 (63.0; 92.0) [81.9 ± 27.5]	76.0 (61.0; 96.0) [80.8 ± 25.8]	0.922
Baseline systolic blood pressure (mmHg), median (IQR) [mean ± SD]	115.0 (101.5; 129.0) [116.2 ± 22.3]	113.0 (100.2; 128.0) [114.9 ± 23.3]	118.0 (103.0; 130.0) [117.4 ± 21.0]	114.0 (99.7; 128.0) [116.2 ± 22.7]	0.400
Baseline mean blood pressure (mmHg), median (IQR) [mean ± SD]	86.0 (76.0; 96.0) [87.8 ± 28.3]	86.0 (76.0; 97.7) [86.5 ± 16.5]	88.0 (78.0; 96.0) [88.1 ± 15.0]	84.0 (73.0; 93.2) [88.6 ± 44.5]	0.181

bpm, beats per minute; IQR, interquartile range; min, minutes; SD, standard deviation.

**Table 3:** Multivariable binary logistic regression analysis evaluating predictors of the primary endpoint.

	Regression Coefficient B	Standard Error	Wald	df	*p*-Value	Odds Ratio (95%CI)
Overweight	−0.035	0.193	0.033	1	0.855	0.965 (0.661–1.410)
Obesity	0.058	0.212	0.075	1	0.784	1.060 (0.700–1.604)
Female gender	0.344	0.172	3.988	1	0.046	1.411 (1.006–1.977)
Age	−0.004	0.008	0.290	1	0.590	0.996 (0.980–1.012)
Single-vessel CAD	0.047	0.256	0.034	1	0.854	1.048 (0.634–1.733)
Two-vessel CAD	−0.430	0.318	1.834	1	0.176	0.650 (0.349–1.212)
Three-vessel CAD	0.360	0.253	2.021	1	0.155	1.434 (0.872–2.355)
Mildly reduced LVEF	−0.010	0.251	0.002	1	0.968	0.990 (0.606–1.618)
Moderately reduced LVEF	−0.213	0.263	0.657	1	0.418	0.808 (0.482–1.353)
Severely reduced LVEF	0.269	0.226	1.419	1	0.233	1.309 (0.841–2.037)
Arterial hypertension	−0.096	0.201	0.230	1	0.632	0.908 (0.612–1.347)
Hyperlipoproteinemia	−0.241	0.172	1.961	1	0.161	0.786 (0.561–1.101)
Diabetes mellitus	0.407	0.225	3.283	1	0.070	1.502 (0.967–2.334)
COPD	0.212	0.358	0.352	1	0.553	1.237 (0.613–2.493)
Smoker	0.325	0.289	1.268	1	0.260	1.384 (0.786–2.437)
Former Smoker	−0.173	0.223	0.603	1	0.438	0.841 (0.543–1.302)

CAD, coronary artery disease; COPD, chronic obstructive pulmonary disease; CI, confidence interval; LVEF, left ventricular ejection fraction.

**Table 4:** Overview of the endpoints between both groups.

	Total (*n =* 726)	Normal (*n* = 236)	Overweight (*n* = 265)	Obesity (*n* = 225)	*p*-Value (Between Groups)
Oxygen saturation <90%, n (%)	308.0 (42.4)	103.0 (43.6)	110.0 (41.5)	95.0 (42.2)	0.575
Hypoxia, n (%)	278.0 (38.3)	95.0 (40.3)	96.0 (36.2)	87.0 (38.7)	0.406
PvCO_2_ > 70 mmHg, n (%)	29.0 (4.0)	10.0 (4.2)	10.0 (3.8)	9.0 (4.0)	0.878
PvCO_2_ > 30% from baseline, n (%)	125.0 (17.2)	39.0 (16.5)	48.0 (18.1)	38.0 (16.9)	0.956
Venous pH < 7.25, n (%)	125.0 (17.2)	40.0 (16.9)	44.0 (16.6)	41.0 (18.2)	0.774
Systolic blood pressure < 80 mmHg, n (%)	278.0 (38.3)	90.0 (38.1)	100.0 (37.7)	88.0 (39.1)	0.809
Mean blood pressure < 65 mmHg, n (%)	372.0 (52.1)	122.0 (51.7)	132.0 (49.8)	118.0 (52.4)	0.504
Midazolam dose (mg), median (IQR) [mean ± SD]	5.0 (5.0; 5.0) [4.9 ± 1.2]	5.0 (5.0; 5.0) [5.0 ± 1.0]	5.0 (5.0; 5.0) [4.7 ± 1.3]	5.0 (5.0; 5.0) [4.9 ± 1.4]	0.309
Midazolam dose (mg/KG), median (IQR) [mean ± SD]	0.06 (0.05; 0.07) [0.06 ± 0.02]	0.07 (0.06; 0.08) [0.07 ± 0.02]	0.06 (0.05; 0.06) [0.06 ± 0.02]	0.05 (0.04; 0.05) [0.05 ± 0.15]	<0.001
Propofol dose (mg), median (IQR) [mean ± SD]	570.0 (360.0; 800.0) [640.5 ± 422.2]	500.0 (333.5; 750.0) [564.0 ± 351.0]	570.0 (380.0; 825.0) [658.6 ± 439.1]	600.0 (390.0; 850.0) [693.0 ± 454.7]	0.002
Propofol dose (mg/KG), median (IQR) [mean ± SD]	6.8 (4.5; 9.6) [7.6 ± 4.7]	7.4 (5.0; 10.5) [8.2 ± 4.8]	6.8 (4.5; 10.0) [7.8 ± 5.0]	6.3 (4.1; 8.8) [6.8 ± 4.0]	0.008
MED Fentanyl i.v. total (mg), median (IQR) [mean ± SD]	0 (0; 16.5) [10.3 ± 15.5]	0 (0; 16.5) [11.2 ± 14.4]	0 (0; 16.5) [9.4 ± 13.9]	0 (0; 16.5) [10.4 ± 18.2]	0.219
MED Fentanyl i.v. (mg/KG), median (IQR) [mean ± SD]	0 (0; 0.2) [0.13 ± 0.20]	0 (0; 0.3) [0.16 ± 0.21]	0 (0; 0.2) [0.11 ± 0.17]	0 (0; 0.2) [0.10 ± 0.20]	0.004
MED Remifentanil i.v. total (mg), median (IQR) [mean ± SD]	3.1 (0; 6.9) [5.5 ± 35.1]	1.6 (0; 6.6) [3.8 ± 5.3]	3.3 (0; 7.3) [7.9 ± 56.9]	3.5 (0; 7.3) [4.3 ± 5.0]	0.423
MED Remifentanil i.v. (mg/KG), median (IQR) [mean ± SD]	0.03 (0; 0.09) [0.06 ± 0.36]	0.02 (0; 0.10) [0.05 ± 0.07]	0.04 (0; 0.09) [0.09 ±0.58]	0.03 (0; 0.07) [0.04 ± 0.05]	0.353

IQR, interquartile range; i.v., intravenous; KG, kilogram of body weight; MED, morphine equivalent dose; PvCO_2_, partial pressure of venous carbon dioxide; pH, potential of hydrogen.

## Data Availability

The data presented in this study are available on request from the authors. The data are not publicly available due to data privacy laws.

## Supplementary Materials

The following supporting information can be downloaded at: https://www.mdpi.com/article/10.3390/healthcare14040517/s1, Supplementary Table S1. Definition and Coding of Variables Included in the Multivariable Regression Models.

## Abbreviations

The following abbreviations are used in this manuscript:

BMI	Body mass index
BP	Blood pressure
BPM	Beats per minute
CAD	Coronary artery disease
CEP	Complex electrophysiological procedure
CI	Confidence interval
CO_2_	Carbon dioxide
COPD	Chronic obstructive pulmonary disease
Df	Degrees of freedom
I.v.	Intravenous
IQR	Interquartile range
KG	Kilogram of body weight
LVEF	Left ventricular ejection fraction
MED	Morphine equivalent dose
Mg	Milligram
Min.	Minutes
OSAS	Obstructive sleep apnea syndrome
PCE	Primary composite endpoint
PvCO_2_	Partial pressure of venous carbon dioxide
pH	Potential of hydrogen
pO_2_	Partial pressure of oxygen
ROC	Receiver operating characteristic
SD	Standard deviation
SpO_2_	Peripheral oxygen saturation
TCM	Transcutaneous CO_2_ monitoring

## References

[1] Van Gelder I.C., Rienstra M., Bunting K.V., Casado-Arroyo R., Caso V., Crijns H.J.G.M., De Potter T.J.R., Dwight J., Guasti L., Hanke T. (2024). 2024 ESC Guidelines for the Management of Atrial Fibrillation Developed in Collaboration with the European Association for Cardio-Thoracic Surgery (EACTS): Developed by the Task Force for the Management of Atrial Fibrillation of the European Society of Cardiology (ESC), with the Special Contribution of the European Heart Rhythm Association (EHRA) of the ESC. Endorsed by the European Stroke Organisation (ESO). Eur. Heart J..

[2] Management of Ventricular Tachycardias: Insights on Centre Settings, Procedural Workflow, Endpoints, and Implementation of Guidelines—Results from an EHRA Survey | EP Europace | Oxford Academic. https://academic.oup.com/europace/article/26/2/euae030/7607829.

[3] Tilz R.R., Busch S., Chun K.R.J., Frerker C., Gaede L., Steven D., Tiefenbacher C., Eckardt L., Sander M., Zwißler B. (2024). Analgosedierung in der Kardiologie: Konsensuspapier der DGK und DGAI 2024. https://www.ai-online.info/archiv/2024/03-2024/analgosedierung-in-der-kardiologie-konsensuspapier-der-dgk-und-dgai-2024.html.

[4] Sairaku A., Yoshida Y., Hirayama H., Nakano Y., Kondo N., Kihara Y. (2014). Don’t Move during Ablation of Atrial Fibrillation!. Int. J. Cardiol..

[5] Huschak G., Busch T., Kaisers U.X. (2013). Obesity in Anesthesia and Intensive Care. Best Pract. Res. Clin. Endocrinol. Metab..

[6] Boutari C., Mantzoros C.S. (2022). A 2022 Update on the Epidemiology of Obesity and a Call to Action: As Its Twin COVID-19 Pandemic Appears to Be Receding, the Obesity and Dysmetabolism Pandemic Continues to Rage On. Metabolism.

[7] Nalliah C.J., Sanders P., Kottkamp H., Kalman J.M. (2016). The Role of Obesity in Atrial Fibrillation. Eur. Heart J..

[8] Kim T.K. (2021). Obesity and Anesthetic Pharmacology: Simulation of Target-Controlled Infusion Models of Propofol and Remifentanil. Korean J. Anesthesiol..

[9] Benson M., Hubers J., Caldis M., Gopal D., Pfau P. (2020). Safety and Efficacy of Moderate Sedation in Super Obese Patients Undergoing Lower and Upper GI Endoscopy: A Case-Control Study. Obes. Surg..

[10] Vargo J.J. (2009). Procedural Sedation and Obesity: Waters Left Uncharted. Gastrointest. Endosc..

[11] Wani S., Azar R., Hovis C.E., Hovis R.M., Cote G.A., Hall M., Waldbaum L., Kushnir V., Early D., Mullady D.K. (2011). Obesity as a Risk Factor for Sedation-Related Complications during Propofol-Mediated Sedation for Advanced Endoscopic Procedures. Gastrointest. Endosc..

[12] Park J.H., Choi S.M., Park J.H., Lee K.H., Yun H.J., Lee E.K., Choi B.M., Noh G.J. (2018). Population Pharmacokinetic Analysis of Propofol in Underweight Patients under General Anaesthesia. Br. J. Anaesth..

[13] Zhu Q., Cui L., Li X., Li Q., Wang Y. (2025). Young, Healthy, Underweight Women Require Higher Effective Doses of Propofol for Successful Gastroscope Insertion: A Dose-Finding Study Using Dixon’s Up-and-Down Method. Drug Des. Dev. Ther..

[14] Bautista A., Hrushka L., Lenhardt R. (2020). Procedural Sedation in the Morbidly Obese: Implications, Complications, and Management. Int. Anesthesiol. Clin..

[15] Sundararaman L., Goudra B. (2024). Sedation for GI Endoscopy in the Morbidly Obese: Challenges and Possible Solutions. J. Clin. Med..

[16] (2018). Practice Guidelines for Moderate Procedural Sedation and Analgesia 2018: A Report by the American Society of Anesthesiologists Task Force on Moderate Procedural Sedation and Analgesia, the American Association of Oral and Maxillofacial Surgeons, American College of Radiology, American Dental Association, American Society of Dentist Anesthesiologists, and Society of Interventional Radiology*. Anesthesiology.

[17] Teumer Y., Buss A., Diofano F., Aktolga D., Katov L., Bothner C., Dahme T., Öchsner W., Mayer B., Rottbauer W. (2024). Prospective Randomized Evaluation of Transcutaneous Carbon Dioxide Monitoring during Complex Electrophysiological Procedures under Deep Sedation: The TRACES Trial. Clin. Res. Cardiol..

[18] Kilic E.T., Sayar S., Kahraman R., Ozdil K. (2019). The Effects of Obesity on Sedation-Related Outcomes of Advanced Endoscopic Procedures. North. Clin. Istanb..

[19] Khan I., Chatterjee A.B., Bellinger C.R., Haponik E. (2016). Sedation for Bronchoscopy and Complications in Obese Patients. Respiration.

[20] Phelps N.H., Singleton R.K., Zhou B., Heap R.A., Mishra A., Bennett J.E., Paciorek C.J., Lhoste V.P., Carrillo-Larco R.M., Stevens G.A. (2024). Worldwide Trends in Underweight and Obesity from 1990 to 2022: A Pooled Analysis of 3663 Population-Representative Studies with 222 Million Children, Adolescents, and Adults. Lancet.

[21] Ahrens M., Rosenthal O., Bau T., Magerfleisch P., Moser F., Frank D., Maslova V., Lian E. (2025). Hemodynamic and Respiratory Stability in Obese vs. Non-Obese Patients during de Novo Pulmonary Vein Isolation under Conscious Sedation. Europace.

[22] Scheurlen C., van den Bruck J.-H., Filipovic K., Wörmann J., Arica Z., Erlhöfer S., Dittrich S., Heijman J., Lüker J., Steven D. (2022). Procedural and Outcome Impact of Obesity in Cryoballoon versus Radiofrequency Pulmonary Vein Isolation in Atrial Fibrillation Patients. J. Interv. Card. Electrophysiol..

[23] Von Thaer S., McVey J., Shelton J., Johnson Q. (2024). Obesity and Anesthesia: Challenges in the Perioperative Period. Mo Med..

[24] Littleton S.W. (2012). Impact of Obesity on Respiratory Function. Respirology.

[25] Maslova V., Ahrens M., Rosenthal O., Bau T., Magerfleisch P., Moser F., Zaman A., Saad M., Spehlmann M., Frank D. (2025). De Novo Pulmonary Vein Isolation in Obese vs. Nonobese Patients under Deep Sedation: Does Obesity Increase Procedure Complexity?. Heart Rhythm O^2^.

[26] Tabaja C., Younis A., Santageli P., Farwati M., Braghieri L., Nakagawa H., Saliba W.I., Madden R., Bouscher P., Kanj M. (2023). Impact of Obesity on Catheter Ablation of Atrial Fibrillation: Patient Characteristics, Procedural Complications, Outcomes, and Quality of Life. J. Cardiovasc. Electrophysiol..

[27] Ingrande J., Lemmens H.J.M. (2010). Dose Adjustment of Anaesthetics in the Morbidly Obese. Br. J. Anaesth..

[28] Abernethy D.R., Greenblatt D.J. (1982). Pharmacokinetics of Drugs in Obesity. Clin. Pharmacokinet..

[29] Hanley M.J., Abernethy D.R., Greenblatt D.J. (2010). Effect of Obesity on the Pharmacokinetics of Drugs in Humans. Clin. Pharmacokinet..

[30] Cortínez L.I., Anderson B.J., Penna A., Olivares L., Muñoz H.R., Holford N.H.G., Struys M.M.R.F., Sepulveda P. (2010). Influence of Obesity on Propofol Pharmacokinetics: Derivation of a Pharmacokinetic Model. Br. J. Anaesth..

[31] Hoymork S.C., Raeder J. (2005). Why Do Women Wake up Faster than Men from Propofol Anaesthesia?. Br. J. Anaesth..

[32] Wasilczuk A.Z., Rinehart C., Aggarwal A., Stone M.E., Mashour G.A., Avidan M.S., Kelz M.B., Proekt A. (2024). Hormonal Basis of Sex Differences in Anesthetic Sensitivity. Proc. Natl. Acad. Sci. USA.

[33] Katov L., Huggle W., Teumer Y., Buss A., Diofano F., Bothner C., Öchsner W., Rottbauer W., Weinmann-Emhardt K. (2025). Gender-Specific Differences in Sedation-Associated Outcomes During Complex Electrophysiological Procedures. Healthcare.

