# Supplementary material for: Impact of Body Weight on Sedation-Related Outcomes in Complex Electrophysiological Interventions
Source: Healthcare (Basel). 2026 Feb 18;14(4):517. doi: 10.3390/healthcare14040517 (PMC12940383; doi:10.3390/healthcare14040517)
Supplement: Supplementary file 1 [file healthcare-14-00517-s001.zip › healthcare-4125942-supplementary.pdf]

Supplementary Table S1. Definition and Coding of Variables Included in the Multivariable Regression Models

Variable	Role in Analysis	Type	Coding / Reference Category
Primary composite endpoint	Dependent variable	Binary	0 = No event; 1 = Event
BMI category – Overweight	Independent variable	Binary (dummy)	1 = Overweight; 0 = otherwise
BMI category – Obesity	Independent variable	Binary (dummy)	1 = Obesity; 0 = otherwise
Normal weight	Reference category	—	BMI_ow = 0 and BMI_ob = 0
Sex	Independent variable	Binary	1 = Female; 0 = Male
Age	Covariate	Continuous	Years
Single-vessel CAD	Covariate	Binary (dummy)	1 = Yes; 0 = No
Two-vessel CAD	Covariate	Binary (dummy)	1 = Yes; 0 = No
Three-vessel CAD	Covariate	Binary (dummy)	1 = Yes; 0 = No
Mildly reduced LVEF	Covariate	Binary (dummy)	1 = Yes; 0 = otherwise
Moderately reduced LVEF	Covariate	Binary (dummy)	1 = Yes; 0 = otherwise
Severely reduced LVEF	Covariate	Binary (dummy)	1 = Yes; 0 = otherwise
Arterial hypertension	Covariate	Binary	1 = Yes; 0 = No
Diabetes mellitus	Covariate	Binary	1 = Yes; 0 = No
Hyperlipoproteinemia	Covariate	Binary	1 = Yes; 0 = No
COPD	Covariate	Binary	1 = Yes; 0 = No
Current smoker	Covariate	Binary (dummy)	1 = Yes; 0 = No
Former smoker	Covariate	Binary (dummy)	1 = Yes; 0 = No
BMI × Sex interaction (Overweight × Female)	Interaction term	Binary	BMI_ow × Female
BMI × Sex interaction (Obesity × Female)	Interaction term	Binary	BMI_ob × Female

BMI, body mass index; CAD, coronary artery disease; COPD, chronic obstructive pulmonary disease; LVEF, left ventricular ejection fraction; ob, obesity; ow, overweight.
